# Non‐invasive Flicker Neurostimulation Mitigates Stress Pathology in a Sex‐Dependent Manner

**DOI:** 10.1002/alz.093441

**Published:** 2025-01-03

**Authors:** Tina Carla Franklin, Matthew Goodson, Hector Zepeda, Nancy Kye, Sara Bitarafan, Jacob Kraus, Luke Braun, Ashley Prichard, Levi B Wood, Annabelle C Singer

**Affiliations:** ^1^ Georgia Institute of Technology, Atlanta, GA USA

## Abstract

**Background:**

Chronic stress promotes life‐long risk for neuropsychiatric decline by increasing neuroinflammation and disrupting synaptic health and plasticity. Our lab and others have recently demonstrated that non‐invasive gamma sensory stimulation (flicker) modulates immune signaling, restores microglial function, and improves cognitive performance in mouse models of Alzheimer’s disease (AD). However, no research to date has studied the effects of flicker in the context of stress. Accordingly, our goal for this study was to determine if flicker mitigates neuropsychiatric‐like behavioral deficits and rescues glial and synaptic pathology following chronic stress.

**Method:**

Daily audiovisual flicker intervention was introduced concomitantly with daily stress exposure (28 days) at multiple frequencies in male and female C57BL6 mice (n = 6/group per sex). The mice were then tested for anxiety‐like behaviors and anhedonia using a range of behavioral tests. We quantified transcriptomic changes in cortical Thy1+ pyramidal neurons, microglia, and astrocytes to identify cell‐type specific genes modulated by flicker intervention in chronically stressed mice. To determine how flicker mitigates stress‐induced cellular changes in the prefrontal cortex, a region vulnerable to stress and AD pathology, we next quantified spine density changes in Thy1‐GFP mice and measured glial morphology changes as a proxy for microglial and astrocyte reactivity using the semi‐automated Imaris imaging software to determine synaptic health and inflammatory profile, respectively.

**Result:**

We found that stress‐induced molecular changes in the prefrontal cortex are modulated in a sex‐, and frequency‐specific manner that coincides with behavioral resilience in stressed mice. Our findings indicates that audiovisual flicker protects against stress‐induced behavioral deficits and mitigates stress‐induced neuroimmune responses in a sex‐ and frequency‐specific manner.

**Conclusion:**

Together, these findings show frequency optimized flicker intervention improves stress pathology and may prevent neuropsychiatric health decline in conditions with sex dimorphic symptoms and prevalence.

